# Response to Yu, Wang, and Ge

**DOI:** 10.1093/jnci/djag213

**Published:** 2026-06-30

**Authors:** Florence Joly, Amélie Anota, Sylvie Chabaud, Claire Cropet, Eric Pujade-Lauraine, Jean-Emmanuel Kurtz, Isabelle Ray-Coquard

**Affiliations:** Centre François Baclesse, University Unicaen, Inserm U1086 Anticipe, Caen, France; Groupe d’Investigateurs Nationaux pour l’Etude des Cancers Ovariens (GINECO), France; Centre Léon Bérard, Lyon, France; Centre Léon Bérard, Lyon, France; Centre Léon Bérard, Lyon, France; ARCAGY Research, Paris, France; Groupe d’Investigateurs Nationaux pour l’Etude des Cancers Ovariens (GINECO), France; Department of Medical Oncology, Hôpitaux Universitaires de Strasbourg, Strasbourg, France; Groupe d’Investigateurs Nationaux pour l’Etude des Cancers Ovariens (GINECO), France; Centre Léon Bérard, University Claude Bernard Lyon I, Lyon, France

We thank Drs Yu, Wang, and Ge for their thoughtful comments^1^ on our PAOLA-1 health-related quality of life (HRQoL) article. In PAOLA-1, it was decided that patients would complete HRQoL questionnaires during planned visits every 12 weeks to coincide with the timing of tumor assessment scans.^2^ We agree that patients receiving poly(ADP-ribose) polymerase (PARP) inhibitor maintenance therapy may experience onset of adverse events such as fatigue, nausea, and vomiting earlier than the 12-week time point.^3^ Given this earlier onset and because the first postbaseline HRQoL assessment in our analysis was at week 12, we agree that there is potential for stabilization bias at 12 weeks.

Reassuringly, results of our PAOLA-1 HRQoL analysis evaluating olaparib plus bevacizumab were consistent with those of SOLO-1^4^ and other trials^5^^,^^6^ demonstrating that PARP inhibitor maintenance monotherapy had no detrimental effect on HRQoL in patients with newly diagnosed advanced ovarian cancer. In SOLO-1, patients with a *BRCA* mutation received olaparib maintenance monotherapy, and HRQoL questionnaires were completed on day 29 in addition to being completed every 12 weeks from baseline, meaning that any impact of adverse events on HRQoL at this earlier time point was captured.^4^ Of the SOLO-1 patients still receiving olaparib maintenance monotherapy at the start of month 2 (*n* = 248) and month 3 (*n* = 242), more than 90% and nearly 85%, respectively, were still receiving the recommended starting dosage of olaparib 300 mg twice daily,^3^ suggesting that a minority of patients receiving olaparib maintenance monotherapy experienced dose reductions to manage adverse events during the first 2 months of therapy. Patients in routine clinical practice should be closely monitored for adverse events during the first few months of PARP inhibitor maintenance therapy, with or without bevacizumab, to optimize adherence.

Data evaluating the impact of disease progression on HRQoL are currently limited, and our exploratory post hoc analysis of HRQoL following disease progression should be regarded as hypothesis generating. Of the 465 patients with RECIST progression during 2 years of follow-up, only 103 (22.2%) completed HRQoL questionnaires following progression and were included in our analysis. Completion of HRQoL questionnaires at 12-week visits and not specifically at the time of progression may have contributed to the missing data, but as our analysis included patients with progression revealed by scan rather than patients with symptomatic progression, rapid physical decline may not have been a major contributor to noncompletion of HRQoL questionnaires. Indeed, analysis of reasons for noncompletion of HRQoL questionnaires in the overall population suggested that “site missed administration” may be a more likely explanation than patients being too ill. Although we cannot exclude a “missing not at random” profile, resulting in an underestimation of the impact of disease progression on HRQoL domains, we believe that our analysis is informative regarding the impact that a diagnosis of progression by scan may have on emotional and social functioning. Further research is needed to better understand the potential HRQoL benefit associated with the delay in both disease progression and subsequent chemotherapy that PARP inhibitor maintenance therapy with or without bevacizumab may provide.

## Data Availability

ARCAGY-GINECO has a long history of academic data sharing for research purposes. The process is similar for every trial sponsored by ARCAGY-GINECO (or ARCAGY Research). Researchers must submit a request to the sponsor directly or through the principal investigator. The request should be written in a predefined format of a short synopsis indicating the objective of the research; the methodology intended to be used, including the statistical analysis plan; and the variables within the database required for the research. A scientific board will review and approve the requests on a case-by-case basis. Only encoded datasets will be used, which enables us to fulfill legal and ethical obligations to protect our patients while at the same time utilizing patient data in progressing medical research to its full potential in the best interests of public health. A specific agreement between the sponsor and the researcher is requested for data transfer. This data transfer agreement details both parties’ responsibilities to ensure the required level of data integrity and legal and ethical obligations. In the case of sharing encoded patient-level data, please note that the full dataset may not be shared in view of the following: clinical consent for some countries prohibits secondary use of the data; patients may withdraw their consent for participation in the trial at any point; and other aspects might also be taken into consideration to protect patient privacy (eg, review of rare clinical events where information is aggregated to a higher level before sharing).
